# Bleaching Bones

**Published:** 1893-05

**Authors:** 


### Bleaching Bones.

Bones may be bleached by placing them in a solution of eleven ounces of caustic soda in forty-five ounces of water; after having been left for two days in this liquid, they are to be well washed in water and immersed in a solution of seventeen ounces of sodium sulphite in forty-five ounces of water. At the end of five or six days, one ounce of hydrochloric acid in five ounces of water must be added to the solution, in which the bones are to be left for a couple of days more, well covered. At the end of that time they are taken out, washed in water and dried.

These quantities of bleaching agents are sufficient for twenty-two ounces of bones.

A very effective method of bleaching bones so as to give them the appearance of ivory, said to have been first used at the Bavarian Museum, is as follows: After digesting the bones with ether or benzine, to remove the fat, they are then thoroughly dried and immersed in a solution of phosphoric acid in water, containing one per cent. of phosphoric anhydride. After a few hours they are removed from the solution, washed in water and dried, when they will have the appearance indicated above.—*Druggists' Circular.*

THE doom of another educational fad is sealed, writes a Paris correspondent. The French association of Volapukists has dissolved. The most energetic apostle of the language which was expected to set right the confusion caused by the affair at Babel has recently taken the post of professor of German in a provincial college. There have been other set-backs, and the great object of reforming the linguistic evils of the world has been abandoned, as far as Paris is concerned.

